# Hyaluronic acid-modified and verteporfin-loaded polylactic acid nanogels promote scarless wound healing by accelerating wound re-epithelialization and controlling scar formation

**DOI:** 10.1186/s12951-023-02014-x

**Published:** 2023-07-26

**Authors:** Kun Chen, Yuanhu Liu, Xiaohui Liu, Yongli Guo, Jing Liu, Jiaojiao Ding, Zheng Zhang, Xin Ni, Yunsheng Chen

**Affiliations:** 1 Department of Burn and Plastic Surgery, Beijing Children’s Hospital, Capital Medical University, National Center for Children’s Health, Beijing, 100045 China; 2 Department of Otolaryngology, Head and Neck Surgery, Beijing Children’ s Hospital, Capital Medical University, National Center for Children’ s Health, Beijing, 100045 China; 3 Shunyi Maternal and Children’s Hospital of Beijing Children’s Hospital, Beijing, China; 4 Beijing Key Laboratory for Pediatric Diseases of Otolaryngology, Head and Neck Surgery, MOE Key Laboratory of Major Diseases in Children, Beijing Pediatric Research Institute, Beijing Children’s Hospital, Capital Medical University, National Center for Children’s Health, Beijing, 100045 China; 5 Department of Plastic and Reconstructive Surgery, Shanghai Ninth People’s Hospital, School of Medicine, Shanghai Jiao Tong University, 639 Zhizaoju Road, Shanghai, 200011 China; 6 Department of Burn, Shanghai Burn Institute, Ruijin Hospital, Shanghai Jiao Tong University School of Medicine, 197 Ruijin 2nd Road, Shanghai, 200025 China

**Keywords:** Hyaluronic acid, Polylactic acid nanoparticles, Scarless wound healing, Verteporfin, Yes-associated protein

## Abstract

Wound healing is a common occurrence. However, delayed healing and aberrant scarring result in pathological wound healing. Accordingly, a scarless wound healing remains a significant clinical challenge. In this study, we constructed hyaluronic acid (HA)-modified and verteporfin (VP)-loaded polylactic acid (PLA) nanogels (HA/VP-PLA) to promote scarless wound healing by accelerating wound re-epithelialization and controlling scar formation. Owing to the unique structure of HA incorporating and coating in VP-loaded PLA nanoparticles, HA/VP-PLA could be topically applied on wound to achieve targeted delivery to fibroblasts. Then, HA/VP-PLA released HA and lactic acid (LA) to stimulate the proliferation and migration of fibroblasts, as well as VP to inhibit Yes-associated protein (YAP) expression and nuclear localization to suppress fibrosis. In vitro (skin fibroblasts) and in vivo (rat and rabbit models) experiments strongly suggested that HA/VP-PLA promoted scarless wound healing by accelerating wound re-epithelialization and controlling scar formation. Therefore, our work provides a feasible strategy for scarless wound healing, and the sophisticated HA/VP-PLA exhibit a great potential for clinical applications.

## Introduction

Wound healing is a common occurrence in humans, and it has been an active research area for many years [1, 2]. It is an extremely complex and coordinated process comprising of four sequential, overlapping biological stages (hemostasis, inflammation, proliferation, and remodeling) where in damaged tissues are restored [3]. Perturbations by external and internal factors in the wound-healing process may can lead to an unsatisfactory outcome, further resulting in delayed healing and aberrant scarring [3]. Therefore, there is an urgent need to develop a scarless wound healing approach targeting delayed healing and aberrant scarring.

The molecular and cellular mechanisms underlying wound healing are extensively studied [4, 5]. However, achieving perfect scarless wound healing remains difficult. In adult humans, scar formation is the result of wound healing, and is characterized by dermal hyperplasia with a dense extracellular matrix (ECM) and disorganized collagen [6]. Recently, the mechanism of scar formation has been reported to be the persistent activation of Yes-associated protein (YAP) that up-regulates fibrosis and leads to excessive ECM deposition [7, 8]. Thus, YAP inhibition could represent a therapeutic strategy to suppress fibrosis in the treatment of various fibrosis diseases [9, 10]. Verteporfin (VP), a Food and Drug Administration (FDA) approved porphyrin compound, has confirmed to inhibit YAP expression, suppress fibrosis and yield scarless skin regeneration [11, 12]. Therefore, VP-based YAP inhibition is a candidate for controlling scar formation, however, suppressed fibrosis prevents wound re-epithelialization [13]. Fortunately, Fortunately, the emergence of nanotechnology integrates the ability to accelerate wound re-epithelialization and control scar formation.

Recently, nanotechnology has provided a new opportunity for wound healing using different strategies to achieve antimicrobial properties, drug delivery, and wound microenvironment regulation [14, 15]. Currently, poly(D,L-lactic acid) (PLA) nanoparticles (PLA NPs) are approved for biomedical applications and as successful modalities for wound healing [16–18]. Furthermore, lactic acid (LA), a product of PLA degradation, is regarded to accelerate wound re-epithelialization by stimulating fibroblast proliferation [19, 20]. Therefore, the combination of PLA NPs and VP can promote scarless wound healing by accelerating wound re-epithelialization and controlling scar formation. However, the application of VP-loaded PLA NPs (VP-PLA NPs) to wounds is challenging.

Nanogel-based topical treatments represent a classic approach for wound healing [21, 22]. Nanogels have a specific hydrophilic 3D macromolecular network structure that offers excellent water-retention properties and colloidal stability [23]. Currently, hyaluronic acid (HA) exhibits excellent performance in nanogel preparations used to promote wound healing by stimulating the proliferation and migration of fibroblasts [24, 25]. HA, a natural polysaccharide composed of N-acetyl glucosamine and D-glucuronic acid in the ECM, is a biologically active, biocompatible, and biodegradable material. In particular, HA-modified nanoparticles have been studied for targeted delivery to fibroblasts, owing to their expression of HA receptors (such as CD44) [26]. Interesting, HA showed the amphiphilic property due to it contained the hydrophobic and hydrophilic patch domain [27]. The hydrophobic patch domain of the CH group facilitates HA to interact with PLA to be incorporated and coated in PLA NPs. With its hydrophilic patch domain, HS could improve the stability of PLA NPs. Furthermore, HA in PLA NPs promote the release of VP from PLA NPs owing to its hydrolytic degradation property [28, 29]. Therefore, HA-modified VP-PLA NP nanogels (HA/VP-PLA) might provide an ideal approach for scarless wound healing by accelerating wound re-epithelialization and controlling scar formation.

**Scheme 1:**
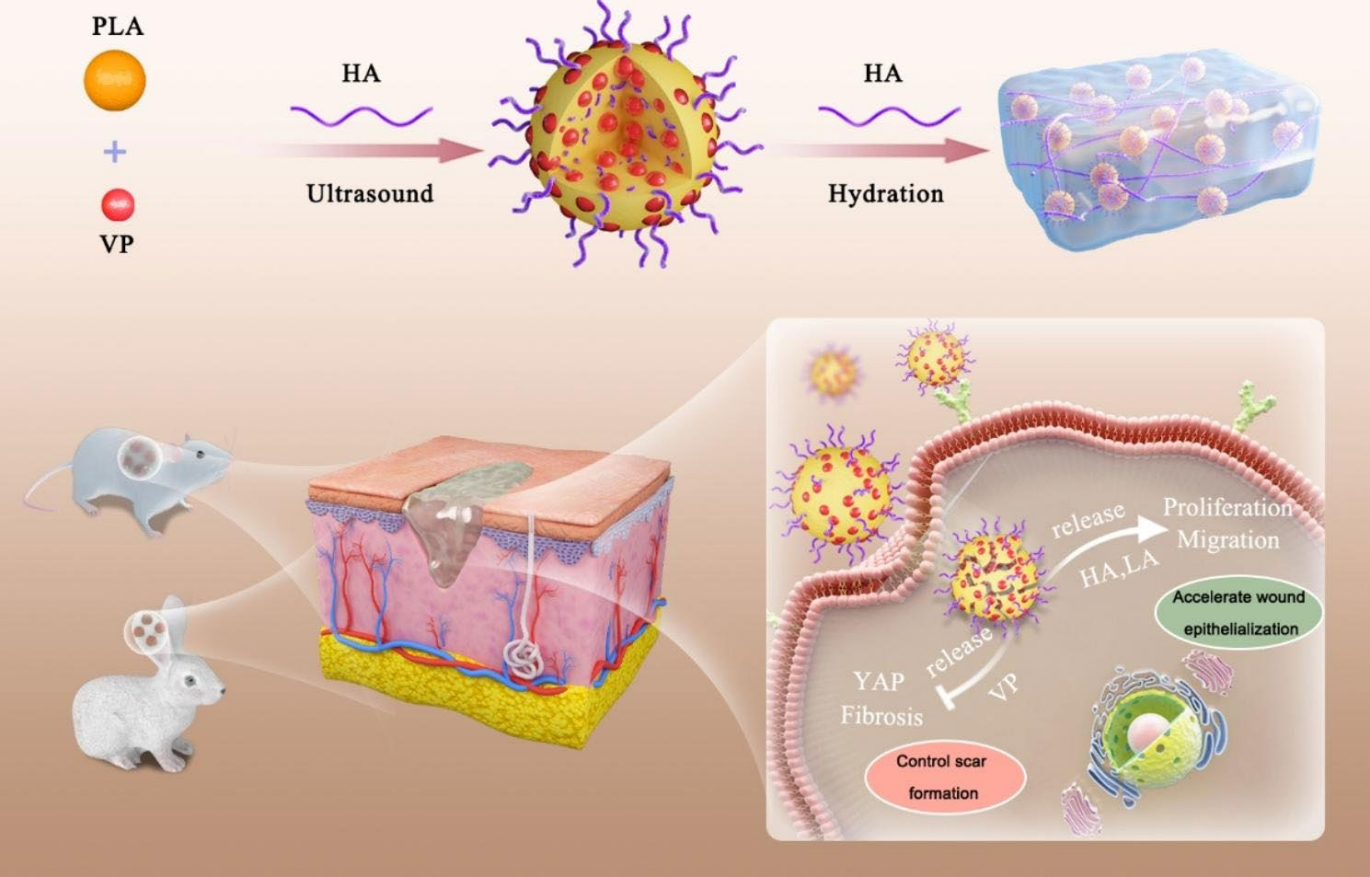
Schematic illustration of HA/VP-PLA promoting scarless wound healing by accelerating wound re-epithelialization and controlling scar formation

Hence, sophisticated HA/VP-PLA, constituting HA-incorporated and HA-coated VP-PLA NPs, were prepared for scarless wound healing. HA/VP-PLA can achieve a targeting delivery to fibroblasts, release HA and LA to accelerate wound re-epithelialization, and realize VP-based YAP inhibition to suppress fibrosis and control scar formation (Scheme 1). HA/VP-PLA exhibited excellent properties to inhibit YAP and stimulate the proliferation and migration of fibroblasts. Histopathological analysis showed that HA/VP-PLA accelerated wound re-epithelialization and controlled scar formation in both rat and rabbit models in vivo. This study thus presents a strategy for scarless wound healing.

## Methods and experiments

### Preparation and characterization

HA/VP-PLA were prepared through nanoprecipitation. Briefly, VP (16 µg, Sigma-Aldrich) and PLA (40 mg, molecular weight 47 kDa with a carboxylic end group, Sigma-Aldrich) in acetone (1 mL) were added gradually into a 10 mL HA (0.5 mg/mL, 1.0 MDa, Aladdin) ethanol aqueous solution (20% ethanol, v/v) with stirring for 2 h. Subsequently, the organic solvent was evaporated on the rotary evaporator at 30 °C, and the aqueous solution was sonicated in an ultrasonic water bath (40 min, 300 W). Finally, HA/VP-PLA were obtained after hydration with HA (80 mg) overnight. VP-free nanogels (PLA-HAs) and VP in the HA nanogels (VP-HAs) were prepared using a similar procedure.

HA/VP-PLA were examined *via* transmission electron microscopy (TEM, JEM-2010, Japan) *via* negative staining with sodium phosphotungstate solution (1.5% w/v, 20 s), cryogenic TEM (Cryo-TEM, Talos F200C G2, USA).and scanning electron microscopy (SEM, JSM-6360LA, Japan). Ultraviolet-visible (UV-Vis) spectra were obtained using a Varian Cary 50 UV-Vis spectrophotometer (Perkin Elmer, USA). The in vitro release of VP was carried out by incubating 500 µL of HA/VP-PLA in 5 mL of PBS with 10% fetal bovine serum (FBS, to mimic physiological conditions) at 37 °C. An aliquot of the sample (50 µL) was used for the fluorescence measurement of the released VP. The entrapment efficiency (EE) and VP release were calculated as follows.

EE = analyzed weight of VP / theoretical weight of VP x 100%.

VP release = total VP released / total VP added x 100%.

Therefore, EE could be considered equal to VP release. Furthermore, the samples were used to determine the PLA-generating LA using high performance liquid chromatography (HPLC) according to a previous studies [30].

### Cell cultures and model constructions

Human skin fibroblasts were isolated from foreskin tissues using a collagenase digestion. The filtrated fibroblasts were collected and grown in Dulbecco’s modified eagle medium containing fetal bovine serum (FBS, 10%), and the culture medium was changed every 3 days. All cells were cultured at 37 °C under 5% CO_2_ in a humidified atmosphere. Cells were used for subsequent experiments after 3–6 generations.

Animal studies were approved by the Animal Experimentation Ethics Committee of the Shanghai Ninth People’s Hospital (IACUC number: SH9H-2021-A428-SB). To verify wound re-epithelialization, eighteen male Sprague Dawley rat (180–250 g) were anesthetized with 2.5% isoflurane, the dorsal area was shaved and disinfected with iodophenol and 75% alcohol, and four full-thickness incisions (10 mm in diameter) were created on both sides of the spine. The formulations were then applied to wound and kept for 30 min every 2 day for 2 weeks. Twelve male New Zealand rabbits (1.8-2 kg) were used to verify the scar formation. Rabbits were anesthetized by baring cartilage on the ventral surface of ear, and four wounds (10 mm in diameter) were created by exposing the cartilage on the ventral surface of ear. The formulations were then applied to the wound and maintained for 30 min every 2 days for the first 2 weeks.

### Cell studies

#### Cell viability detection

Fibroblasts were seeded in 96-well plates (2,000 cells per well) for 24 h. Then, the culture medium was replaced with FBS-free medium and fresh formulations were added (the same equivalent concentrations) for 24 h. Cells were then incubated with Cell Counting Kit-8 (CCK-8) for 4 h, and optical density was measured using a microplate reader at a wavelength of 450 nm. Cell viability was expressed as the percentage, which was normalized to the value of the control.

#### Cellular uptake

Fibroblasts were seeded in glass-bottom dishes for 24 h. The culture medium was then replaced with FBS-free medium containing the different formulations, and the cells were incubated for 6 h. The cells were imaged using a confocal laser scanning microscopy system (CLSM, Leica TCS SP5, Germany) with VP fluorescence (425 nm excitation, 690 nm emission) and 4’,6-diamidino-2-phenylindole (DAPI) fluorescence (405 nm excitation, 488 nm emission) to determine cellular uptake and localization. In addition, fibroblasts treated with HA/VP-PLA were collected, standard methods for TEM were carried out, and the cellular uptake of HA/VP-PLA was observed.

#### YAP inhibition, fibrosis, proliferation and migration of fibroblasts

Fibroblasts were seeded on 1 cm coverslips for 8 h, and then incubated with medium containing formulations for 24 h. Cells were fixed with paraformaldehyde and permeabilized with Triton-100. The coverslips were incubated sequentially with the indicated primary antibodies overnight and fluorescein isothiocyanate (FITC)-conjugated secondary antibodies at room temperature for 2 h. Finally, the antibody localization and cell structures were visualized using CLSM. Furthermore, the fibrosis-related proteins (TGF-β1 and α-SMA) and a proliferation-related protein (PCNA) were analyzed *via* western blot analyses. A scratch assay was performed for cell migration. The scratch was created perpendicular to the back of the horizontal line using a vertically positioned pipette tip, and fibroblasts were cultured with medium containing formulations for 48 h.

### Animal studies

#### Assessment of wound re-epithelialization in rat models

Digital images of the wounds were captured every 4 days to analyze wound re-epithelialization. the rats were euthanized with an overdose of sodium pentobarbital (150 mg/kg), and tissues were harvested for histological evaluations after 16 days. Masson and Sirius red staining were used in histopathological analysis. The ratio of red/green area in Sirius red staining images were quantified using ImageJ (version 1.48). Meanwhile, immunofluorescence analysis of YAP, TGF-β, α-SMA and MMP-3 was conducted using the respective antibodies.

#### Assessment of scar formation in rabbit models

After 30 days of post-surgery, the rabbits were euthanized with an over-dose of pentobarbital sodium, and tissues were prepared for histological evaluations. Tissue sections were made across the most elevated portion of the scar and stained with Hematoxylin-eosin (HE), Masson and Sirius red staining. Scar elevation index (SEI, the ratio of the scar height to the normal skin height) was used to evaluate scar formation. Meanwhile, immunohistochemical staining for TGF-β1, α-SMA, collagen I, and collagen III was conducted using respective antibodies. The sections were examined, and immune-positive cells were scanned and counted using a scanner system (ScanScope XT, Aperio, CA).

### Statistical analysis

The results are expressed as the mean ± standard deviation. Statistical analysis was performed by performing Student’s t-tests using Origin with *p* < 0.05 as the minimal level of significance.

## Results and discussions

### Characterization studies

**Fig. 1:**
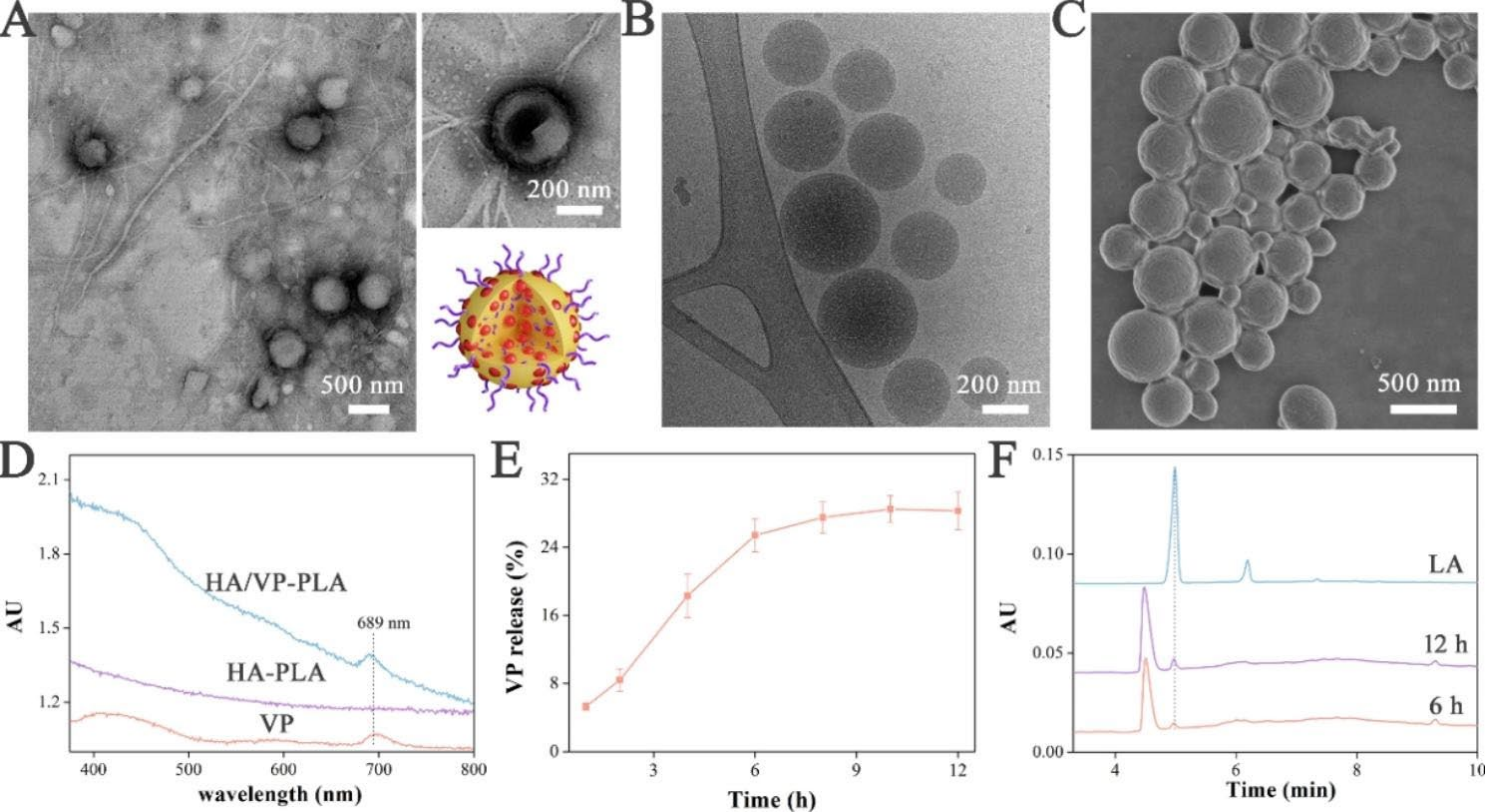
** A**: TEM image of HA/VP-PLA with detail and schematic; **B-C**: Cryo-TEM and SEM images of HA/VP-PLA; **D**: UV-Vis spectra (the absorption peak of VP was 689 nm); **E**: Release profiles; **F**: HPLC spectra of LA detection (LA: LA standard, 6 and 12: PLA degradation for 6 and 12 h)

The morphological features were initially studied using electron microscopy. Spherical HA/VP-PLA were embedded in visible HA meshes, and HA chains incorporated into and coated VP-PLA NPs according to TEM images (Fig. 1A). Cryo-TEM images showed that HA/VP-PLA comprised of homogenous nanoparticles and their structure was favorable for loading hydrophobic VP owing to the interaction between hydrophobic VP and hydrophobic PLA (Fig. 1B). Furthermore, well dispersion indicated that the amphipathicity of HA could improve the stability of HA/VP-PLA. Scanning electron microscopy images showed that spherical HA/VP-PLA were distributed in the HA gel matrix (Fig. 1C). This endowed HA/VP-PLA with sufficient adhesion for direct application to the wound to maintain a moist environment, and sustainably produce effective components.

The physicochemical features, including VP loading and degradation of HA/VP-PLA, were also extensively studied. UV-Vis spectroscopy revealed that VP was successfully loaded onto HA/VP-PLA (Fig. 1D). Subsequently, the degradation of HA/VP-PLA, involving VP release and LA generation, was studied by measuring VP and LA. Most of loaded VP (28.3 ± 2.2% of dosage) was release in 12 h. Therefore, EE of VP in HA/VP-PLA, considered equal to VP release, was satisfactory (Fig. 1E). Compared with that in previously reported work, HA/VP-PLA had a faster release rate (6 h vs. 40 h), because the structure of the HA incorporated in PLA NPs could promote the disaggregation of NPs to release VP with its hydrolytic degradation property [31]. As expected, The LA content increased with time (6 and 12 h), verifying that PLA degradation could generate LA as expected (Fig. 1F).

In summary, the prepared HA/VP-PLA had a unique structure and physicochemical features that facilitated targeted delivery of VP, HA and LA to improve scarless wound healing.

### Cell studies in vitro

#### Cellular biocompatibility

**Fig. 2:**
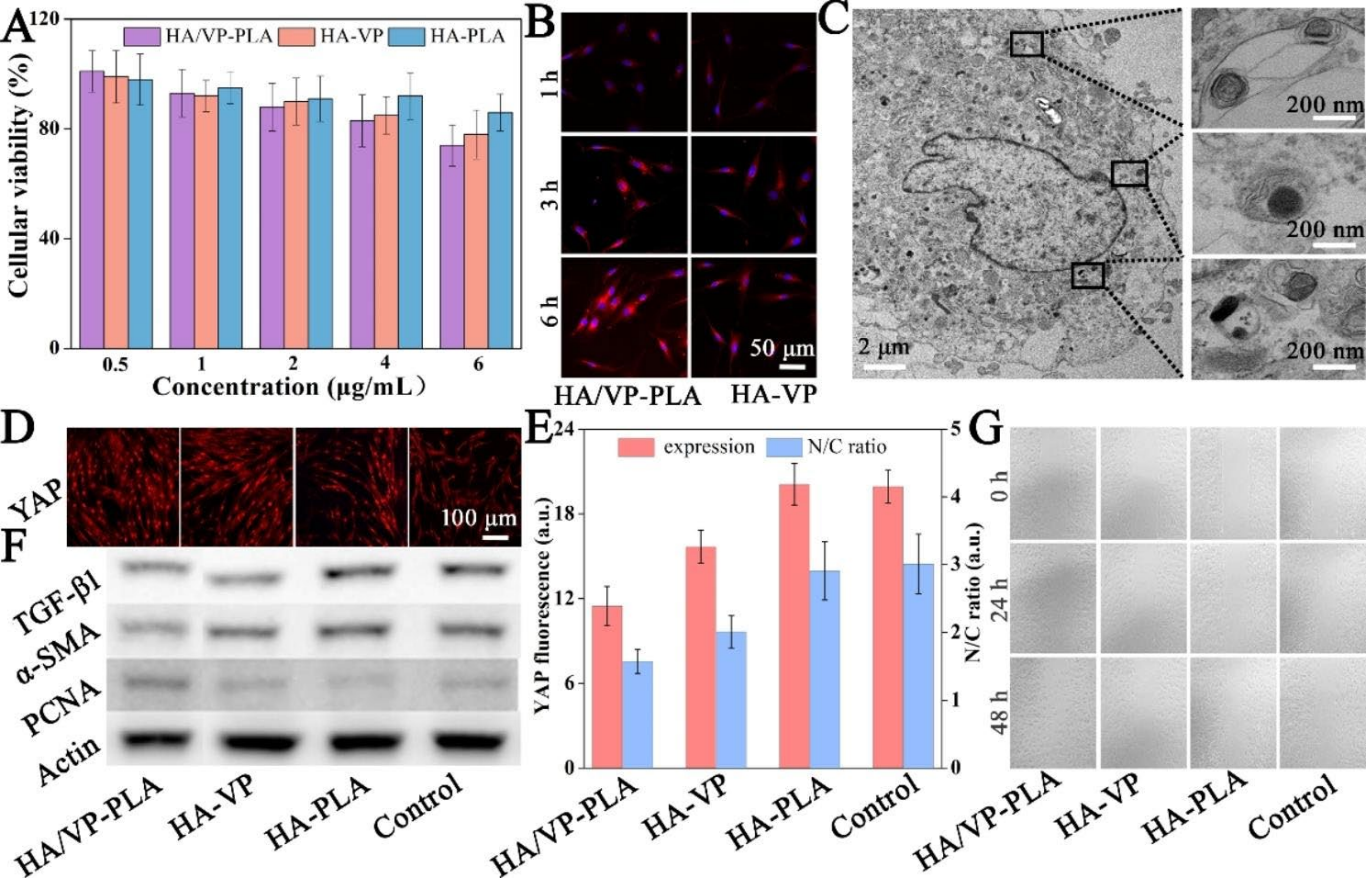
** A**: The cell viability of fibroblasts treated by formulations; **B**: CLSM images of VP delivered into fibroblasts; **C**: Ultrastructural observation of fibroblasts treated with HA/VP-PLA; **D**: CLSM images of YAP expression; **E**:Statistics analysis of the YAP expression and nuclear localization; **F**: fibrosis and proliferation-related protein expressions; **G**: scratch assay

The biocompatibility of HA/VP-PLA is crucial for their potential applications, and a greater cellular uptake of HA/VP-PLA increases YAP inhibition as well as cytotoxicity. All formulations exhibited concentration-dependent cytotoxicity according to CCK-8 assays (Fig. 2A). However, cytotoxicity (cellular viability > 90%) was insignificant at low VP concentrations (< 2 µg/mL). HA/VP-PLA and HA-VP had 85–90% of the cellular viability at 4 and 6 µg/mL, respectively. However, there was a slight decrease in viability (< 85% of the cellular viability) at 8 µg/mL. In contrast, HA-PLA did not affect cell viability at any concentration (cellular viability > 90%). Therefore, HA/VP-PLA had excellent biocompatibility, and 2 µg/mL VP was used for downstream cell studies to balance its cytotoxicity and YAP inhibition.

#### Cellular uptake studies

The cellular uptake of HA/VP-PLA was evaluated using VP fluorescence. Compared to that in VP-HA group, HA/VP-PLA group showed much stronger fluorescence, depicting cell profiles, and the fluorescence intensity increased with the incubation time (Fig. 2B). Furthermore, HA/VP-PLA resulted in some intense red fluorescent dots on the cytomembrane and cytoplasm, suggesting that abundant HA/VP-PLA actively targeted the cell surface by utilizing HA receptors and more efficiently entered the cells, as expected. Furthermore, TEM showed that HA/VP-PLA displayed homogeneous darkness with contrast enhancement emerging inside of endosomes (Fig. 2C). This indicated that HA/VP-PLA readily entered into cell through their enclosure in endosomes [32]. Then, HA/VP-PLA mediated endosome degradation to facilitate the release of VP, HA, and LA inside the fibroblasts.

#### YAP inhibition, fibrosis and proliferation/migration

YAP expression and nuclear localization were studied in the treated fibroblasts to initially determine whether HA/VP-PLA treatment resulted in YAP inhibition and the suppression of fibrosis [33]. Immunostaining analysis of treated fibroblasts showed that HA/VP-PLA treatment significantly inhibited YAP expression (Fig. 2D). Quantification of the YAP fluorescence nucleocytoplasmic intensity ratio indicated that HA/VP-PLA were most efficient at inhibiting nuclear accumulation and improving the cytoplasmic retention of YAP (Fig. 2E). This matched with cellular uptake studies. Furthermore, PLA components did not affect YAP inhibition. Fibrosis suppression was then studied using fibrosis biomarkers. Western blot revealed HA/VP-PLA exhibited the best performance in suppressing fibrosis, based on reduced expression of TGF-β1 and α-SMA (Fig. 2F). Subsequently, western blotting and cell scratch assays were used to assess proliferation and migration ability, two important roles in wound healing. HA/VP-PLA had the best performance in terms of PCNA expression and migration ability of fibroblasts (Fig. 2F and G). Therefore, HA/VP-PLA could inhibit YAP to suppress fibrotic activity, and deliver HA and LA to stimulate the proliferation and migration, which was favorable for scarless wound healing.

### Assessment of wound re-epithelialization in rat models in vivo

**Fig. 3:**
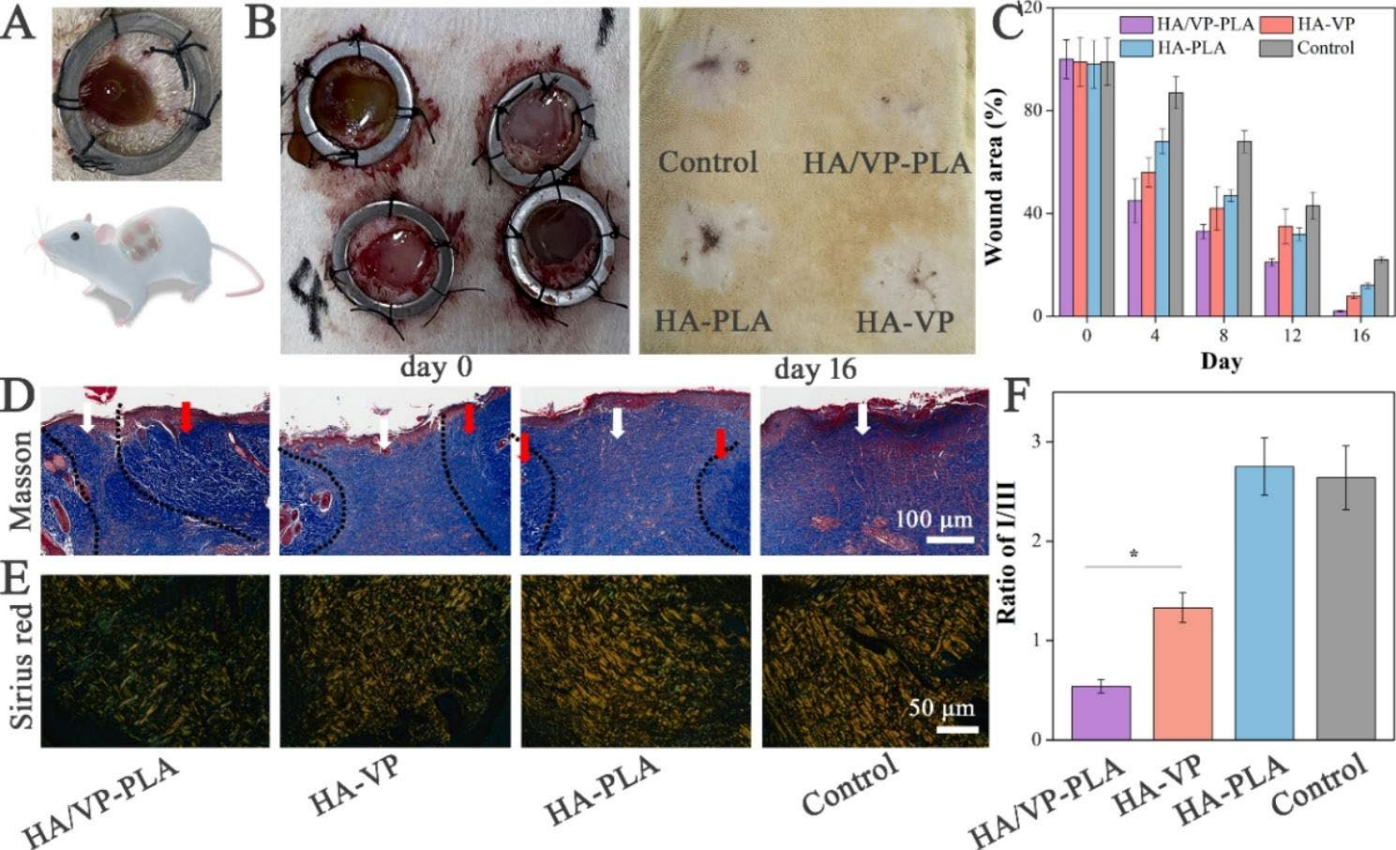
** A**: Schematic of the application of HA/VP-PLA in rat models; **B**: wound re-epithelialization in rat models at the day 0 and day 16r; **C**: Percentage of wound area in each group over 16 days; **D-E**: Masson and Sirius red staining staining of tissues (the area of white arrows: the new-born collagen, the area of red arrows: the mature collagen); F: statistical analysis of the ratio of collagen I to collagen III (*: *p* < 0.05)

HA/VP-PLA were found to promote wound re-epithelialization in rat models. To eliminate individual differences as possible, four wounds in each rat were divided into formulation and control groups, and nanogels were applied to the wounds as shown in schematic presentation (Fig. 3A). Formulations significantly improved epidermal regeneration compared with that in the control group, and HA/VP-PLA exhibited the best performance in terms of wound re-epithelialization, owing to the release of HA and LA (Fig. 3B C).

To evaluate the potential for fibrosis suppression, we analyzed the wound tissue samples in 16 days after wounding. In Masson staining result, formulations promote the significantly more collagen deposition for accelerating wound re-epithelialization. Meanwhile, the collagen remodeling, including new-born collagen converting to mature collagen and reducing collagen I/III ratio, were studied in Masson and Sirius red staining. HA/VP-PLA promoted collagen remodeling involving the conversion of new-born collagen (the area of white arrows) into mature collagen (the area of white arrows) according to Masson’s staining, which improved the mechanical strength of skin [34]. Then, Sirius red staining was also used to evaluate collagen I (yellowish-red) and collagen III (green) in the tissues (Fig. 3E). Control group presented a collagen disorder arrangement with collagen I dominance. In contrast, HA/VP-PLA exhibited the best performance not only in improving the collagen regular arrangement but also increasing the collagen III content, based on a quantification analysis of the collagen I/III ratios (Fig. 3F). Therefore, HA/VP-PLA accelerated and improved wound re-epithelialization.

**Fig. 4:**
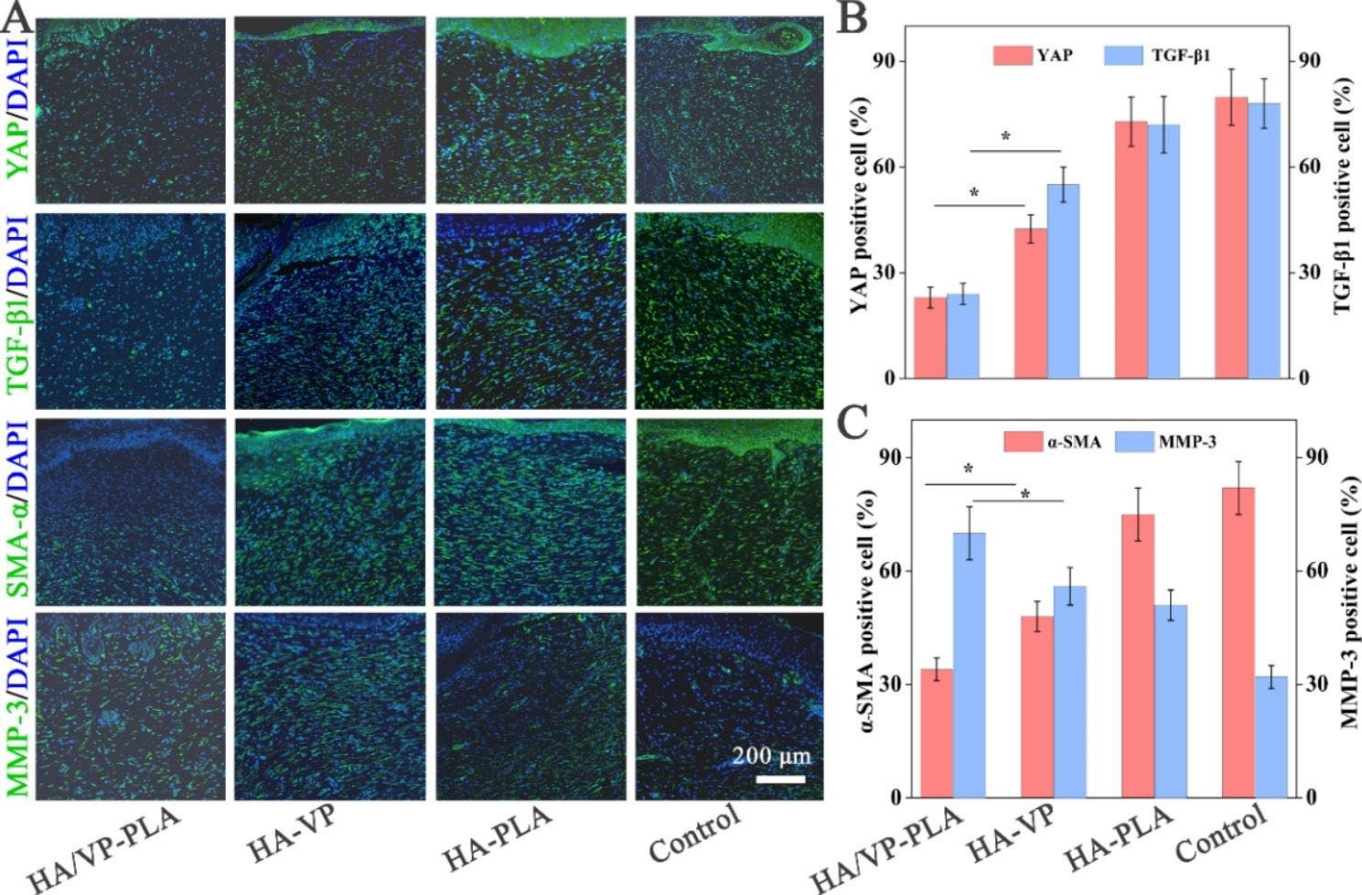
** A**: immunofluorescence analysis of YAP expression and fibrosis biomarkers; **B**: Statistical analysis of YAP and TGF-βα-SMA (*: *p* < 0.05); **C**: Statistical analysis of α-SMA and MMP-3 (*: *p* < 0.05)

YAP inhibition and fibrosis suppression were further demonstrated using in vivo immunofluorescence analysis. HA/VP-PLA exhibited the best performance in terms of YAP inhibition compared with other formulations (Fig. 4A). Furthermore, the expression of TGF-β, α-SMA, and MMP-3 (affecting collagen production and metabolism) proportionally decreased and increased, respectively to YAP inhibition (Fig. 4B C). Therefore, HA/VP-PLA inhibited YAP and suppressed fibrosis owing to its targeted delivery ability, which helped promote scarless wound healing.

### Assessment of scar formation in rabbit models in vivo

**Fig. 5:**
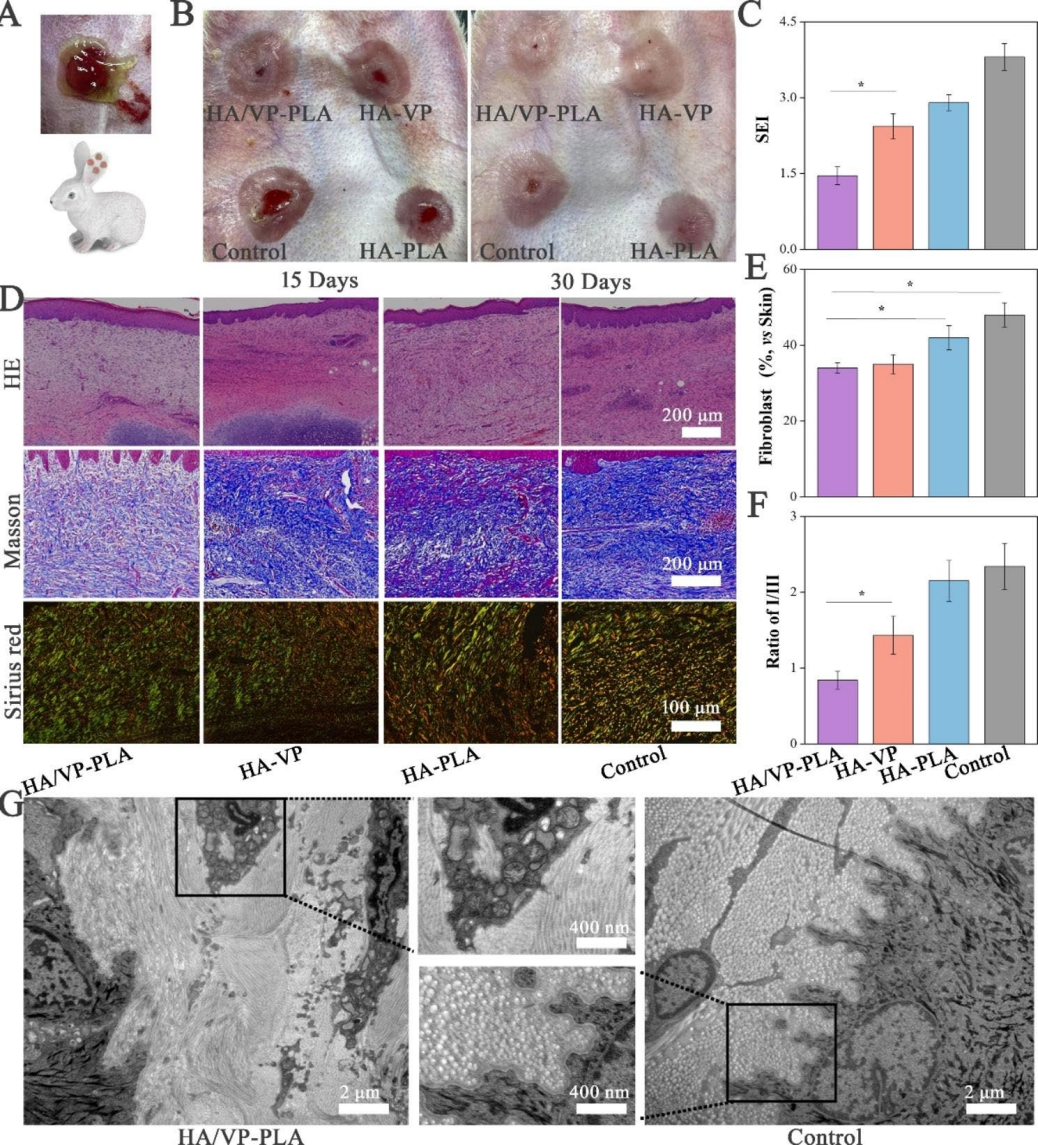
** A**: Schematic of the application of HA/VP-PLA in rabbit models; **B**: The appearance changes of wound tissue at 15 and days post-surgery; **C**: Statistics of SEI of tissues(*: *p* < 0.05); D:HE, Masson and Sirius red staining of tissues; **E**: Statistics of fibroblast proliferation(*: *p* < 0.05); **F**: statistical analysis of the ratio of collagen I to collagen III (*: *p* < 0.05); **G**: Ultrastructural differences of tissue in HA/VP-PLA and control groups

Although rat models provide information on wound re-epithelialization, they were not used to assess scar formation owing to the lack of mechanical tension [35]. Thus, HA/VP-PLA were applied to rabbit ear wound models to further evaluate their potential in controlling scar formation (Fig. 5A). HA/VP-PLA promoted wound epithelialization in rabbit models within 15 days post-surgery, similar to that in rat models. All tissues were thick, and presented a dark red color after 30 days post-surgery; the HA/VP-PLA treatment produced the best morphological appearance (Fig. 5B). The SEI was used to evaluate scar thickness and provide accurate evidence that HA/VP-PLA could significantly control scar formation (Fig. 5C). Fibroblast proliferation and collagen deposition, indicators of scar formation, were assessed *via* histopathological analyses (Fig. 5D). Hematoxylin and eosin (HE) staining analysis for fibroblast proliferation revealed that the HA/VP-PLA group exhibited a remarkable reduction in fibroblasts compared to the umbers in the other groups (Fig. 5E). This phenomenon was explained by the fact that HA/VP-PLA promoted the transition from the proliferation stage to the remodeling stage [36].

Masson staining analysis for collagen deposition and HA/VP-PLA showed significantly reduced collagen fiber deposition and improved collagen arrangements with slender structures. Sirius red staining was further used to evaluated collagen I and collagen III in tissues, and HA/VP-PLA were found to improve the collagen arrangement with collagen III dominating. Statistical analysis of I/III ratios indicated that HA/VP-PLA were more effective in reducing the ratio, as expected (Fig. 5F). Moreover, the slender collagen in HA/VP-PLA group displayed a “basket-weave” pattern and regular arrangement according to TEM, whereas the Control group had a disorganized dense and bulky collagen arrangement (Fig. 5G). Therefore, the ultra-features of collagen were in accordance with scarless wound healing [37].

**Fig. 6:**
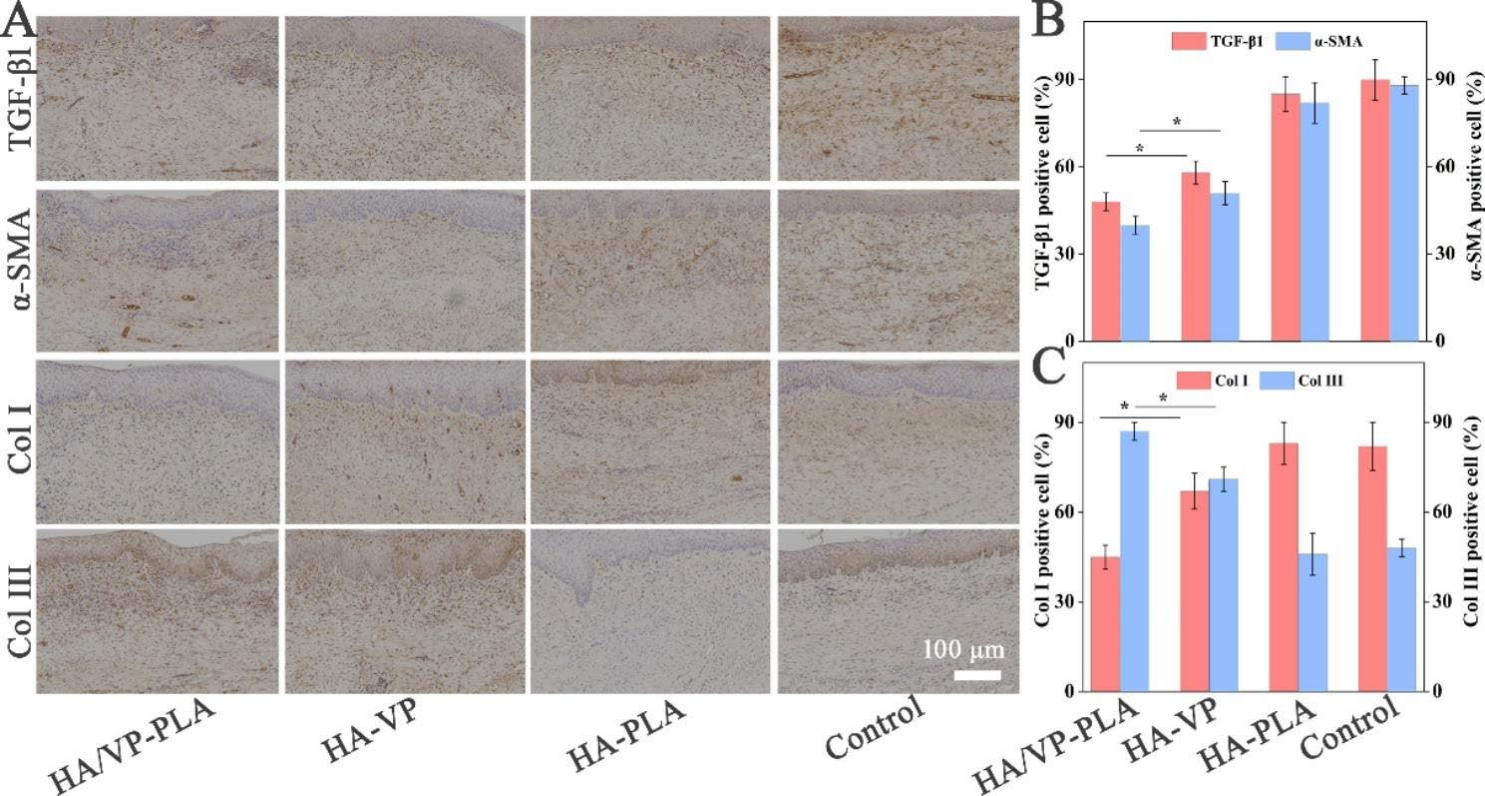
** A**: Immunohistochemical analysis of fibrosis biomarkers; **B: S**tatistical analysis of TGF-β1 and α-SMA (*: *p* < 0.05); **C**: **S**tatistical analysis of collagen I and III (*: *p* < 0.05)

Fibrosis-related protein expression was further studied using immunohisto-chemical analysis (Fig. 6A). The results indicated that HA/VP-PLA had the best performance in suppressing the expression of TGF-β and α-SMA (Fig. 6B). HA/VP-PLA showed improved efficacy in reducing collagen I deposition and augmenting collagen III deposition (Fig. 6C). Therefore, rabbit model studies confirmed that HA/VP-PLA showed great potential to control scar formation by suppressing fibrosis.

Together, these animal studies strongly suggested that HA/VP-PLA could effectively promote scarless wound healing by accelerating wound re-epithelialization and controlling scar formation *via* targeted delivery to inhibit YAP, suppress fibrosis, and stimulate the proliferation and migration of fibroblasts.

## Conclusion

Sophisticated HA/VP-PLA was developed to realize targeted delivery to fibroblasts in wound tissue. They promoted scarless wound healing by accelerating wound re-epithelialization and controlling scar formation. The sustained release of HA and LA was found to stimulate the proliferation and migration of fibroblasts to accelerate wound re-epithelialization. Meanwhile, HA/VP-PLA successfully controlled scar formation, as delivery of VP for YAP inhibition suppressed fibrosis. Detailed studies using fibroblasts and animal models demonstrated the ability of HA/VP-PLA to promote scarless wound healing. Thus, this study provided an effective therapeutic strategy to promote scarless wound healing. Future studies will be performed to provide an additional experimental basis to investigate the clinical applications of HA/VP-PLA.

## Data Availability

The datasets used and/or analyzed during the current study are available from the corresponding author on reasonable request.
